# Graphical modeling of cortical tau pathology topography for its subtyping in Alzheimer’s disease

**DOI:** 10.1162/IMAG.a.1110

**Published:** 2026-01-22

**Authors:** Jiaxin Yue, Xinkai Wang, John Ringman, Yonggang Shi

**Affiliations:** USC Mark and Mary Stevens Neuroimaging and Informatics Institute, Keck School of Medicine, University of Southern California, Los Angeles, CA, United States; Ming Hsieh Department of Electrical and Computer Engineering, Viterbi School of Engineering, University of Southern California, Los Angeles, CA, United States; Department of Neurology, Keck School of Medicine, University of Southern California, Los Angeles, CA, United States; Alfred E. Mann Department of Biomedical Engineering, Viterbi School of Engineering, University of Southern California, Los Angeles, CA, United States

**Keywords:** tau, topography, heterogeneity, Alzheimer’s disease, Reeb graph

## Abstract

Hyperphosphorylated tau tangles are essential hallmarks of Alzheimer’s disease (AD) and their propagation across brain regions was often considered to follow the classic Braak stages. Recent post-mortem and *in vivo* tau positron emission tomography (PET) studies, however, revealed the frequent presence of tau pathology heterogeneity. Clustering or event-based methods were proposed previously for the subtyping to tau pathology in AD, but they often lack robustness to varying distributions of disease severity across cohorts. To robustly discover and model tau pathology subtypes in AD, we propose in this work a novel graphical modeling framework that can disentangle the phenotypical differences of tau PET imaging due to disease heterogeneity from the spatiotemporal variations of disease stages. First, we propose a novel Reeb graph representation at the individual level to characterize the topographic patterns of salient tau pathology on cortical surfaces. Next, we use only cross-sectional tau PET data to develop a graphical model at the population level to encode the inter-subject spatiotemporal relationships, which enables us to robustly derive subtypes based on the topographic patterns of tau pathology and hence achieve increased generalization power to new samples with distinct tau pathology severity from the training data. Using synthetic and large-scale tau PET imaging data from the Alzheimer’s Disease Neuroimaging Initiative (ADNI) and Anti-Amyloid Treatment in Asymptomatic Alzheimer’s (A4) studies, we compare with the state-of-the-art SuStaIn method and demonstrate the improved generalization performance of the proposed method. In addition, we validate both methods on a cohort of autosomal dominant Alzheimer’s disease (ADAD) patients with known tau pathology patterns to show that our method has more robust performance in testing data with large deviations from training data. Furthermore, for preclinical patients of the A4 cohort, we demonstrate more significant differences in clinical cognitive measures can be observed across subtypes discovered by our method.

## 1 Introduction

Tau pathology is one of the defining hallmarks of Alzheimer’s disease (AD) and a key component of the AT(N) framework for AD research and diagnosis (Jack Jr et al., 2016). Compared with the amyloid-β (A) pathology and neurodegeneration (N), tau pathology (T) has much stronger correlation to clinical symptoms (Nelson et al., 2012; Ossenkoppele et al., 2016). The propagation of tau pathology across brain regions was typically considered to follow the classic Braak stages (Braak & Braak, 1991). Recent neuropathological (Murray et al., 2011; Petersen et al., 2019) and *in vivo* tau PET imaging studies (Ossenkoppele et al., 2020; Xia et al., 2017), however, showed the frequent presence of heterogeneity in tau pathology. Using *in vivo*, cross-sectional tau PET data, we propose in this work a novel graphical modeling framework for subtyping tau pathology in AD by characterizing its spatiotemporal topography on cortical surfaces.

The classic Braak stages (Braak & Braak, 1991) describe the progression of tau tangles from the trans-entorhinal cortex to the limbic area, and finally to the whole neocortex. However, emerging evidence from various postmortem and *in vivo* tau PET imaging studies (Murray et al., 2011; Whitwell et al., 2012, 2018) demonstrated the existence of atypical neurofibrillary tangle distributions in AD patients. A medial temporal lobe (MTL)-sparing phenotype as well as a limbic-predominant phenotype has been described in many discoveries (Murray et al., 2011; Vogel et al., 2021; Whitwell et al., 2018). In addition, related clinical variants have been noted for quite a long time that include the posterior cortical atrophy (PCA) (Crutch et al., 2012) and the behavior variant of AD (Ossenkoppele et al., 2015). Recent histology and PET imaging studies have confirmed that these clinical variants exhibit distinct patterns of tau pathology (Nelson et al., 2012; Ossenkoppele et al., 2016; Petersen et al., 2019), highlighting the importance of disentangling tau pathology heterogeneity for better profiling, diagnosis, and targeted treatment of AD.

For the subtyping of tau PET data, clustering-based methods are among the most popular and have been successfully applied to discover distinct tau pathology patterns (Jeon et al., 2019; Lowe et al., 2018; Whitwell et al., 2018). A fundamental limitation of previous clustering-based analysis, however, is ignoring the temporal progressions of imaging patterns across different disease stages (Habes et al., 2020). As a result, an event-based method, Subtype and Stage Inference (SuStaIn) (Young et al., 2018), which can leverage cross-sectional data to model the spatiotemporal heterogeneity of biomarkers, has gained popularity recently and has been successfully applied to a series of studies (Aksman et al., 2023; Mastenbroek et al., 2024; Vogel et al., 2021; Young et al., 2023). In essence, the SuStaIn model assumes the monotonic progression of pathological patterns based on *a set of predefined events* on a small number of selected anatomical areas, which could constrain its ability in representing varying disease trajectories and hence generalization performances in testing cohorts with significantly different distributions of tau pathology severity than the training data.

To capture the spatiotemporal progression patterns while discovering the heterogeneity of tau pathology in AD, we propose a novel graphical modeling framework to analyze tau PET data with improved robustness to variations in imaging feature distributions across cohorts. First, we develop a Reeb graph representation of the topographic patterns of tau pathology on the cortex at the individual level. Next, a graphical model is proposed at the population level for representing the spatiotemporal pattern similarities between subjects that are robust across different cohorts. Finally, subtyping of tau pathology can be achieved via community detection in this graph. Using synthetic and large-scale real tau PET data from the Alzheimer’s Disease Neuroimaging Initiative (ADNI) (Mueller et al., 2005), and Anti-Amyloid Treatment in Asymptomatic Alzheimer’s (A4) (Sperling et al., 2014) studies, we conduct a series of experiments to demonstrate the much improved generalization performance of the proposed method than the SuStaIn method. In addition, we validate both methods on a cohort of autosomal dominant Alzheimer’s disease (ADAD) patients (Ringman et al., 2023) with known tau pathology patterns to show that our method has more robust performance on testing data with imaging feature distributions deviating significantly from training data. Furthermore, for preclinical patients of the A4 cohort, we show that more significant differences in clinical cognitive measures can be observed between the subtypes extracted by our method than those from the SuStaIn method.

A preliminary version of this work has been published in a conference paper (Yue & Shi, 2023). Compared with our conference publication, we have made substantial improvements here to the graphical modeling algorithm including the normalization of the cross penalty function and the graph pruning process. In addition, a more detailed description of all technical steps has been provided. Furthermore, all experimental results are novel that include in-depth comparisons with the SuStaIn method, validations on the ADAD data, and clinical cognitive measures of the preclinical subjects in the A4 study. Finally, an open-source implementation of the proposed method is distributed freely to the research community: (https://github.com/Jiaxin-Yue/Tau-PET-Graphical-Model).

## 2 Materials and Methods

### 2.1 Datasets

Tau PET images (n = 720) from the Alzheimer’s Disease Neuroimaging Initiative (ADNI) (Mueller et al., 2005), Anti-Amyloid Treatment in Asymptomatic Alzheimer’s (A4) (Sperling et al., 2014), and an in-house cohort of ADAD patients are used. All these studies received approval from their respective institutional review boards (IRBs) or ethics committees at each participating site. All participants, along with their study partners when applicable, provided written informed consent prior to data collection, which included consent for data sharing and future research use. The data used in this analysis were fully de-identified to protect participant confidentiality.

Among all the data, 427 amyloid-positive-tau-positive (A+T+) cross-sectional tau PET images from ADNI and A4 cohorts were selected for our experiments on constructing the graphical representation and discovering the subtypes. A+/A- labels were provided by the ADNI and A4 study, and T+/T- status was defined based on whether the peak standardized uptake value ratio (SUVR) of the tau PET image exceeds 1.5 (Jack Jr et al., 2017, 2018). Besides, 279 cognitively normal A-T- participants from the ADNI and A4 cohorts consist of the normal control samples for our experiments. The demographic information of the ADNI and A4 data are listed in Table 1. Additionally, 14 T+ tau PET cross-sectional images from a cohort of ADAD patients are used to further evaluate the clustering stability and subtype patterns. The ADAD subject recruitment and data acquisition were approved by the institutional review board (IRB) at the University of Southern California (USC). Unlike late-onset AD (LOAD), these ADAD patients all carry the A431E mutation in the PSEN1 gene and are predetermined to develop dementia at a similar age range (Murrell et al., 2006). In addition, patients with ADAD tend to present atypically high tau accumulation in parietal lobes (Gordon et al., 2019) instead of medial temporal lobes as in typical LOAD.

**Table 1.** Demographic information of A4 and ADNI subjects.

Cohort	Characteristics	Control	A+T+
A4	Age	69.65 ± 4.29	72.89 ± 4.80
	Gender (M/F)	23/32	115/153
	Diagnosis	55 CN	268 CN
ADNI	Age	73.14 ± 6.02	78.15 ± 6.58
	Gender (M/F)	95/129	78/81
	Diagnosis	224 CN	63 CN/ 17 SMC62 MCI/ 17 AD

CN: cognitive normal; SMC: subjective memory complaint; MCI: mild cognitive impairment; AD: Alzheimer’s disease.

### 2.2 Preprocessing of tau PET data

All imaging data were preprocessed using the standard PETSurfer pipeline (Greve et al., 2014) in FreeSurfer (Fischl et al., 2004). T1-weighted MRIs were first processed by FreeSurfer to obtain their anatomical segmentation using the Desikan–Killiany atlas. Besides, all tau PET images were averaged across frames and registered to the corresponding T1-weighted MRI of the same subject to obtain the skull-stripped tau PET images. The PETSurfer pipeline was then applied to the registered tau PET images for partial volume correction and intensity normalization using the inferior cerebellar gray matter as the reference region to create the resulting tau SUVR images. The volume-based SUVR images were projected onto cortical surfaces of both hemispheres and then a hemisphere symmetric fsaverage_sym atlas (Greve et al., 2013). The regional z-scores were calculated based on the mean and standard deviation of the A-T- normal controls, which will be used by the SuStaIn method (Vogel et al., 2021; Young et al., 2018) for subtyping experiments and comparisons.

In alignment with the Braak staging of tau pathology in AD (Braak & Braak, 1991), which integrates information across hemispheres to define the cortical distribution of tau pathology across different disease stages, we also combine data from two hemispheres of each subject by mapping the SUVR maps of both hemispheres to a common template, the fsaverage_sym atlas in the right hemisphere, and calculate the average SUVR map on this atlas surface as the input to our method and the conversion to z-scores used by the SuStaIn method in all subtyping experiments. This ensures that we can compare the subtyping and staging results directly with the cortical areas involved in different Braak stages.

### 2.3 Reeb graph analysis of tau PET patterns

In this work, we use the tau PET SUVR function (Schöll et al., 2016) on cortical surfaces for heterogeneity analysis. In previous studies (Vogel et al., 2021), researchers generally used average SUVR values of tau PET within a small number of predefined regions of interest (ROIs) as features for studying tau pathology heterogeneity. We propose here to use the Reeb graph of smooth tau SUVR functions to more accurately extract tau pathological patterns for modeling the spatiotemporal relations across subjects. Given a Morse function f on a surface ℳ, its Reeb graph is defined as follows (Reeb, 1946):

**Definition 1.** *Let f:* ℳ → ℝ *be a function defined on the surface* ℳ*. The Reeb graph* R(f) *of* f *is the quotient space with its topology defined through the equivalent relation* x≃y *if* f(x)=f(y) *for* ∀x,y∈ℳ *.*

**Fig. 1.**
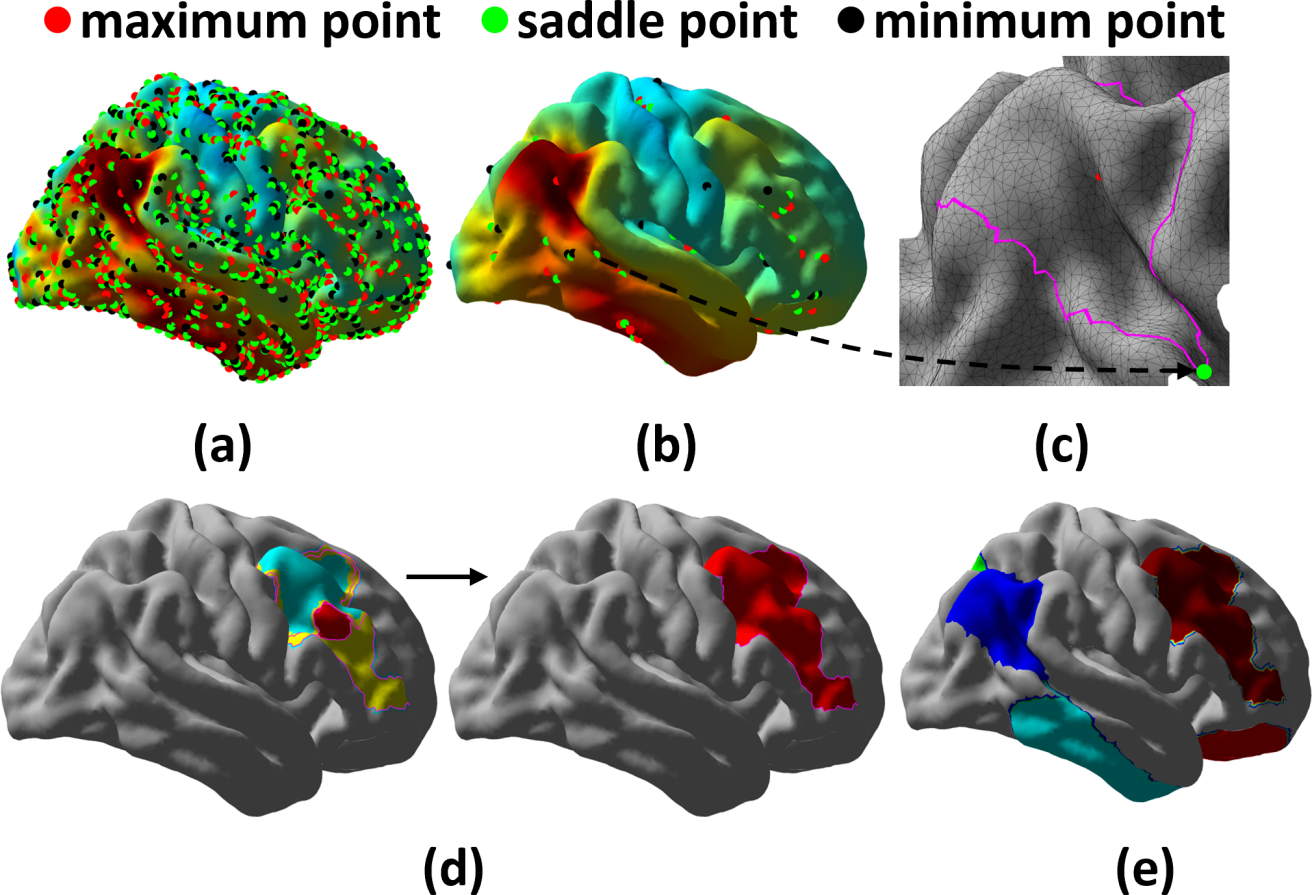
Reeb graph construction and simplification. (a) The original SUVR map contains noisy critical points. (b) After Laplacian smoothing, the Reeb graph is reduced to a common level of complexity (<100 critical points) for all subjects. (c) A level contour (purple curve) in the neighborhood of a saddle point. (d) An illustration of Reeb graph simplification: three edges, represented by patches in different colors, are merged into one edge. (e) The final partition of the surface with the Reeb graph.

In essence, the Reeb graph of a function f is a graph of its critical points and characterizes the topographic distribution of these critical points on the cortical surface. For the numerical implementation of Reeb graph analysis, we use the tau SUVR map f defined on a triangular mesh ℳ common to all subjects, which is downsampled from the fsaverage_sym template of FreeSurfer (Greve et al., 2013) and has around 20,000 vertices. For the numerical calculation of the Reeb graph R(f) , we employ the algorithm proposed in Shi et al. (2012), which was developed for functions on cortical surfaces with genus zero topology. To be more specific, the topology of the Reeb graph only changes at critical points (minimum, maximum, and saddle points) of the function f. For a given SUVR map f on the cortical surface ℳ, we first sort its critical points $C=\{C_{1},C_{2},\cdots ,C_{K}\}$ according to their SUVR values such that $f(C_{1})<f(C_{2})<\cdots <f(C_{K})$ . The surface can be partitioned by the level contours of f in the neighborhood of these critical points (Fig. 1c). Edges connecting these critical points in the Reeb graph are obtained by applying region growing on the mesh ℳ (Shi et al., 2012). The generated Reeb graph is represented as R(f)=(C,E), where C is the nodes of the graph, corresponding to the critical points, and E is the set of edges connecting neighboring nodes. Each edge can be considered as a patch consisting of triangles between the two level contours associated with the neighboring nodes in the graph.

A Reeb graph simplification scheme is further developed to remove the noisy peaks in the original SUVR function, thereby improving the detection of salient patterns. First, we iteratively apply Laplacian smoothing to the SUVR map f until its Reeb graph reaches a common level of complexity (Fig. 1a, b), which is measured as the number of nodes in the graph. Subsequently, we use a Reeb graph simplification algorithm to merge nodes around saddle points based on their persistence (Shi et al., 2014). For an edge $E_{k}=(C_{i},C_{j})(f(C_{i})<f(C_{j}))$ of the Reeb Graph R(f)=(C,E) , we assign its weight based on a persistence measure defined as

$$w(E_{k})=A_{k}\times f(C_{j}),$$ (1)

where $A_{k}$ is the area of the edge $E_{k}$ that connects $C_{i}$ and $C_{j}$, and $f(C_{j})$ is the peak SUVR value of this edge. To simplify the Reeb graph, we iteratively remove saddle points and spurious edges based on the persistence threshold δ. At each iteration, we scan these saddle points and their connected edges sequentially according to their SUVR values, and for an edge $E_{k}=(C_{i},C_{j})$ with $w(E_{k})<\delta$ , we collapse this edge by removing the node $C_{i}$ with smaller SUVR value and adding all its connections to the node $C_{j}$ with a higher SUVR value (Fig. 1d). The weights of these new edges are updated according to Eq. (1). These steps are repeated until the persistency threshold is reached. The number of critical points in the pruned Reeb graph is determined by the complexity of the SUVR function and the persistence threshold, which is set as δ=300 in this work so the pruned Reeb graph typically has less than 10 edges. As illustrated in Figure 1e, patches enclosed by each edge of the pruned Reeb graph successfully capture the salient tau pathology. By reducing the noisy SUVR functions from tau PET imaging to this compact Reeb graph representation, we can more effectively characterize the topographic patterns of tau pathology and model the spatiotemporal relations across subjects as will be described next.

### 2.4 Population-level graph construction based on tau topography similarity

To model the spatiotemporal similarity of the tau pathology between subjects, we develop a graph representation at the population level by measuring the spatial and temporal coherence of the Reeb graph patches of tau SUVR functions from different subjects. Given a query subject x and reference subject y, we first establish correspondences of their Reeb graph patches from x to y based on spatial proximity on the template surface fsaverage_sym . Let $x_{i}$ and $y_{j}$ denote the Reeb graph patches of the tau SUVR functions of x and y, respectively. A distance matrix C is constructed, with each entry $C_{i,j}$ representing the distance between patches $x_{i}$ and $y_{j}$, as follows:

$${\text{C}}_{i,j}=dPeak_{x_{i},y_{j}}\times dHausdorff_{x_{i},y_{j}},$$ (2)

where dPeak is the geodesic distance between the peaks of the two patches, and dHausdorff is the Hausdorff distance between the two patches. If $min_{j}({\text{C}}_{i,j})<c$ , where c is a uniform unpaired cost (typically chosen as 1,000 in our work), the reference patch $y_{j}$ in y is assigned as the corresponding patch to the query patch $x_{i}$ in x based on spatial proximity, and we add the pair $\left\{x_{i},y_{j}\right\}$ to a correspondence set Φ. Otherwise, the query patch $x_{i}$ is designated as unpaired. In this assignment, each query patch $x_{i}$ could have at most one matching patch in y, while for the reference patch $y_{j}$, it could be assigned to multiple patches in x.

**Fig. 2.**
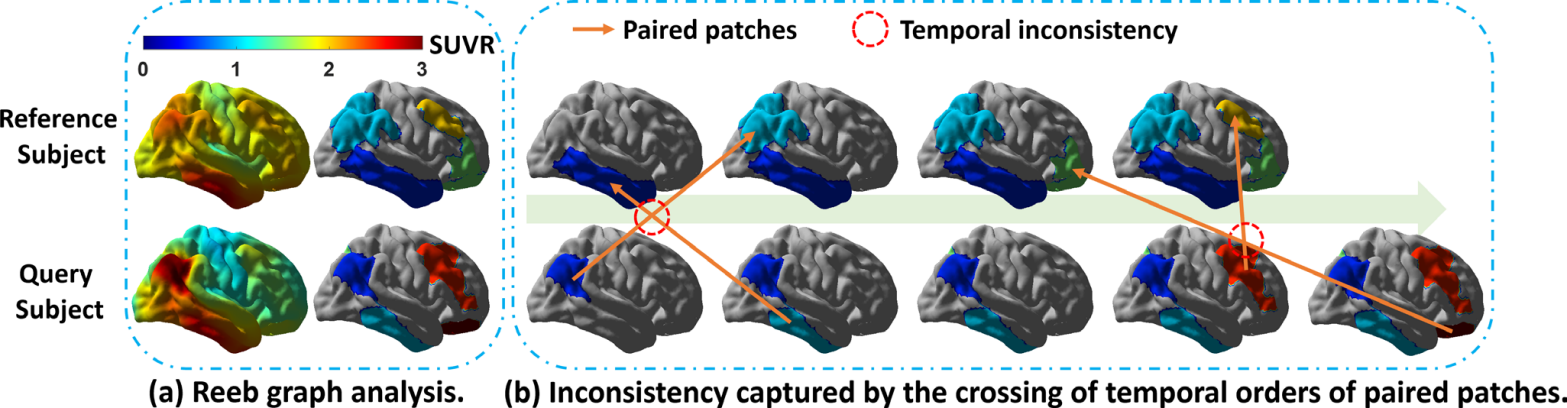
An illustration of the temporal inconsistency measure used for the construction of the proposed population-level graph. (a) Reeb graph analysis is applied to the cortical SUVR map (left column) to extract the topographic patterns of tau pathology (right column). (b) Patches from the reference and query subjects are ordered in decreasing SUVR values (the green arrow) within each subject. For two sets of paired patches from the query and reference subject, they are temporally inconsistent if the patch with the higher SUVR value from the query subject is paired to the patch with the lower SUVR value from the reference subject. This crossing of temporal orders will be recorded with a crossing penalty in our spatiotemporal distance calculation between the two subjects.

Based on the assumption about the monotonic spreading of tau pathology with increasing disease severity (Jack Jr et al., 2010), we propose here the *key idea* to measure the spatiotemporal coherence of subjects by quantifying the temporal inconsistency of their Reeb graph patch correspondences established via spatial proximity on the template mesh (Fig. 2). By sorting the Reeb graph patches based on the peak SUVR value of each patch, we can rearrange the indices of the Reeb graph patches $x_{i}$ for the query subjects x and $y_{j}$ for the reference subject y in a decreasing order of SUVR values, respectively. Given a set of patches from the query subject x, their paired patches in y are temporally consistent if the relative temporal orders of the corresponding query patches in x are preserved. Otherwise, the temporal inconsistency between x and y can be quantified as the crossing of the temporal orders between two tau propagation sequences as illustrated in Figure 2b. A crossing penalty $Dcrossing_{x,y}$ is then defined as

$$\begin{array}{cccc} D_{\text{crossing}}\left(x,y\right) & =\sum_{\left\{x_{i_{1}},y_{j_{1}}\right\}\in \Phi }\sum_{\left\{x_{i_{2}},y_{j_{2}}\right\}\in \Phi }\frac{{\left[SUVR_{x_{i_{2}}}-SUVR_{x_{i_{1}}}\right]}^{+}}{{\text{max}}_{x_{i_{1}}}SUVR_{x_{i_{1}}}}\times & & \\ & I\left(SUVR_{y_{j_{1}}}-SUVR_{y_{j_{2}}}\right)\times \left(A_{x_{i_{1}}}+A_{y_{j_{1}}}\right), & & \end{array}$$ (3)

where Φ is the set containing the corresponding patch pairs $\left\{x_{i_{1}},y_{j_{1}}\right\}$ and $\left\{x_{i_{2}},y_{j_{2}}\right\}$ , $SUVR_{\left(\;\right)}$ denotes the peak SUVR value of a patch, and $A_{\left(\;\right)}$ denotes the size of a patch, and

$$\begin{array}{ccc} I\left(t\right)=\{\begin{array}{cc} 1 & \text{if }t>0\text{;}\\ 0 & \text{otherwise} \end{array}\text{and}\;\;{\left[t\right]}^{+}=\{\begin{array}{cc} t & \text{if }t>0\text{,}\\ 0 & \text{otherwise.} \end{array} & & \end{array}$$

This crossing penalty is normalized by the maximum SUVR value of the subject to reduce the impact of extreme values and encourage the model focusing on pattern changes instead of SUVR deviations across regions. The crossing penalty is designed under the general monotonic assumption of tau accumulation over disease progression and serves as a regularization term in constructing our graphical representations. Rather than strictly forbidding any ordering inconsistencies between subjects, it encourages overall consistency in regional progression patterns while still allowing reasonable flexibility for inter-individual variability and small deviations arising from noise or imaging artifacts.

Besides the temporal inconsistency of the spatially matched Reeb graph patches from subjects x and y, we augment the inconsistency measure by defining a cost for unpaired patches that are not included in the correspondence set Φ as follows:

$$Dunpair_{x,y}=\sum_{x_{i}}\frac{SUVR_{x_{i}}}{{\text{max}}_{x_{i}}\;SUVR_{x_{i}}}\times A_{x_{i}}\times \left(1-1_{i}\right),$$ (4)

where $1_{i}$ is an indicator function defined as

$$\begin{array}{ccc} 1_{i}=\{\begin{array}{cc} 1 & \text{if }\exists y_{j}\;\text{s.t.}\;\left\{x_{i},y_{j}\right\}\in \Phi \text{;}\\ 0 & \text{otherwise.} \end{array} & & \end{array}$$

This unpaired penalty is also normalized by the maximum SUVR value of the subject x.

The total spatiotemporal distance between two subjects x and y is finally calculated as the weighted sum of the two penalty terms

$$D_{x,y}=\alpha \times Dcrossing_{x,y}+Dunpair_{x,y}.$$ (5)

We typically set α=2 to balance the contribution from these two penalty terms. From the distance matrix D, a symmetric distance matrix $\bar{D}$ is constructed as ${\bar{D}}_{x,y}={\bar{D}}_{y,x}=min(D_{x,y},D_{y,x})$ to encourage stronger connectivity between similar subjects. From this, a similarity matrix is derived as $Sim=1/(1+\bar{D})$ and serves as the basis for constructing an undirected population-level graph with the matrix entries acting as the edge weights.

### 2.5 Graph pruning

To represent the temporal relationships among subjects and reduce the ambiguity within dense connections, the fully connected undirected population-level graph is converted into a sparse directed graph by firstly assigning edge directions, and followed by further refinement through edge cutting.

#### 2.5.1 Pairwise directionality

The directionality of edges in the graph could represent the temporal order between subjects, and is estimated from the relative severity of pathology between two subjects. The evaluation of the relative severity for the query subject x based on the reference subject y involves the comparison of the SUVR and size of all tau pathology patches, and can be formulated as

$$\begin{array}{c} M_{x,y}=\sum_{i=1}^{N}{\left[SUVR_{x_{i}}-thd_{pos}\right]}^{+}\times A_{x_{i}}\times \left(1-1_{i}\right)\\ \;\;\;\;\;\;\;\;\;\;\;\;\;\;\;\;+\sum_{\left\{x_{i},y_{j}\right\}\in \Phi }{\left[SUVR_{x_{i}}-SUVR_{y_{j}}-thd_{diff}\right]}^{+}\times A_{x_{i},} \end{array}$$ (6)

where

$$\begin{array}{c} 1_{i}=\{\begin{array}{cc} 1 & \text{if }\exists y_{j}\;\text{s.t.}\;\text{\{}x_{i},y_{j}\text{\}}\in \Phi \text{;}\\ 0 & \text{otherwise,} \end{array} \end{array}$$

$\{x_{i},y_{j}\}\in \Phi$ denotes the corresponding patch pair from the subject x to y, $A_{x_{i}}$ is the patch size, and 1-1 indicates the unpaired patches in x. Two thresholds are used for enhancing the robustness of severity measurement: $thd_{pos}$ is used for identifying the tau positive patches, $thd_{diff}$ is applied for including only patch pairs with meaningful SUVR differences. In this work, we set $thd_{diff}=0.5$ and $thd_{pos}=1.3$ according to Jack Jr et al. (2017) and Weigand et al. (2022). Similarly, we could obtain $M_{y,x}$ by switching the role of x and y.

Between two subjects, higher SUVR values and/or wider spread of pathology indicate a later disease stage. Therefore, by comparing $M_{x,y}$ and $M_{y,x}$ and taking a threshold Δm , we establish three possible edge directions to define a directed graph $S\bar{i}m$ based on the similarity matrix Sim : (1) if $M_{x,y}-M_{y,x}>\Delta m$ , x is at a later stage with $S\bar{i}m_{y,x}=Sim_{y,x}$ and $S\bar{i}m_{x,y}=0$ ; (2) if $M_{y,x}-M_{x,y}>\Delta m$ , y is at a later stage with $S\bar{i}m_{x,y}=Sim_{x,y}$ and $S\bar{i}m_{y,x}=0$ ; (3) if $\left|M_{x,y}-M_{y,x}\right|<\Delta m$ , both subjects are at the same disease stage with $S\bar{i}m_{x,y}=S\bar{i}m_{y,x}=Sim_{x,y}$ . The resulting directed population-level graph $S\bar{i}m$ encodes the pattern similarity and temporal orders through edge weights and directions. We use Δm=1,000 across all experiments, generating the directed population-level graph predominantly composed of uni-directional edges for the subsequent staging, which is elaborated in Section 2.6.

#### 2.5.2 Sparse directed graph for clustering

To improve the computational efficacy and clustering performance, the population-level graph is further pruned to a sparse graph to eliminate the redundant connections and overlapping between communities (Blondel et al., 2008; Wickramasinghe & Muthukumarana, 2022). The K-nearest-neighbor (KNN) approach is used to manage the sparsity by retaining only K edges with the highest similarity for each node and removing the rest connections. With an appropriate choice of K, such as K=15 in all our experiments, KNN effectively transforms the directed graph $S\bar{i}m$ into a sparse directed graph. $S\tilde{i}m$ This transformation preserves the single connected component structure while introducing sufficient sparsity to delineate community boundaries. Additionally, the proportion of uni-directional edges remains steady to support the staging of disease severity.

### 2.6 Clustering and staging of tau pathology

With the sparse directed population-level graph $S\tilde{i}m$ , we employ the efficient Louvain community detection method (Blondel et al., 2008) for graph clustering, which was developed for modular organization extraction through maximizing modularity in network science. In our problem setting, the high modularity can be found when overall similarity within the same subtype is high, and the connections between different subtypes are sparse in the graph. Because the Louvain community detection method is a greedy algorithm and initialization affects the clustering results, we iterate it 100 times to get a consensus matrix. Each element of this matrix represents the frequency of two subjects clustered into the same subtype, and the community detection algorithm with the same setting is applied to this consensus matrix to generate the stable subtyping results. The only parameter required for the Louvian community detection algorithm is the resolution γ to control the module size, and is selected based on our observations of the community distributions in the graphs.

The population-level disease stage within each subtype is derived from the overall directionality of the directed graph $\tilde{Sim}$. In graph theory, the in-degree of a node in the directed graph is defined as the number of edges directed into the node, and the out-degree is defined as the number of edges directed out of this node. For a late-stage subject, whose connections are mostly in earlier stages and the associated edges point toward this subject in the graph, resulting in higher in-degree than out-degree for this node. Conversely, for an early-stage subject, its out-degree is higher than the in-degree. Hence, we could define the relative degree of a node as

$$d\left(v\right)=deg^{-}\left(v\right)-deg^{+}\left(v\right),$$ (7)

where $deg^{-}(v)$ and $deg^{+}(v)$ denote the in-degree and out-degree of the node v, respectively. By thresholding this relative degree, we could assign discrete stages to subjects within each subtype similar to the Braak staging process.

### 2.7 Tau pathology subtyping on new data

Given the tau SUVR data from a training population, we construct a directed population-level graph to represent the spatiotemporal distribution among training subjects, and perform graph-based subtyping to derive the individual subtype memberships. For new samples from a testing dataset, the subtypes can be inferred from the trained graph by applying the K-nearest-neighbor (KNN) method. Specifically, the spatiotemporal distances between the new data and all training data are calculated according to Eqs. (3), (4), and (5), and converted into the similarity measurements. Subtyping membership for the new data is assigned via a majority voting based on the subtypes of the K most similar training subjects. We take the identical K=15 used for graph pruning in all experiments.

This inference is robust across populations because of the normalization in the measurement of the spatiotemporal distances between subjects, which ensures that the KNN method estimates the subtype membership based on pattern similarity and thus minimizes the impact of tau SUVR severity differences. In our experiments, we will demonstrate that this approach offers significant generalization advantages by providing stable subtype inference across varying disease stages and data distributions.

### 2.8 Construction of synthetic data

**Fig. 3.**
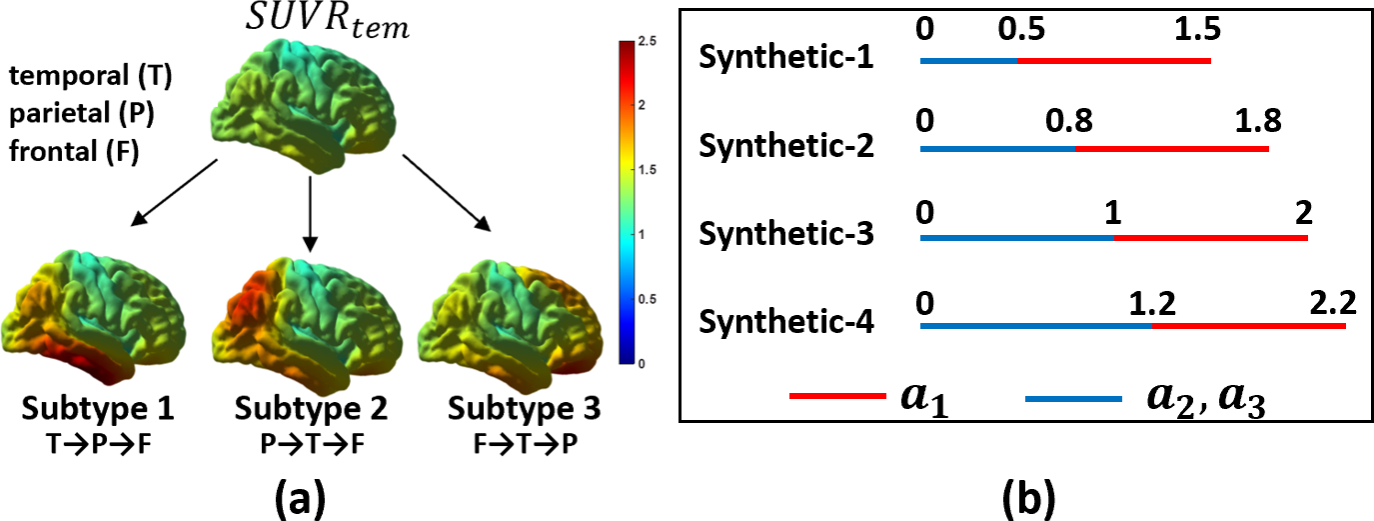
Synthetic data generation. (a) The baseline SUVR map and the average SUVR maps of the three subtypes. The propagation order of the seeds (T: temporal; P: parietal; F: frontal) is listed below each subtype that is consistent with the decreasing order of tau pathology severity levels. (b) For each simulated dataset, the sampling range for seed magnitudes ($a_{1}$: red; $a_{2},a_{3}$: blue).

Evaluating subtyping performance is challenging without ground truth, making synthetic data with predefined patterns and subtypes valuable for quantitative assessments. Based on the tau seeding mechanism (Holmes et al., 2014; Vogel et al., 2020), we generated the synthetic SUVR data by simulating the spread of tau pathology from seeds to other vertices on the surface fsaverage_sym . By averaging the SUVR maps from the normal controls of the ADNI and A4 cohorts, we computed the baseline SUVR map $SUVR_{tem}$ . As shown in Figure 3a, three subtypes were simulated by varying the propagation order, and hence severity levels of different brain regions based on $SUVR_{tem}$ . For each synthetic subject, we randomly sampled a seed vertex in the temporal, parietal, and frontal lobe, respectively. Given the subtype (Fig. 3a) of the current subject, we denote the magnitude of the 1st, 2nd, and 3rd seed as $a_{1}>a_{2}>a_{3}$, respectively, and sampled them randomly according to the range shown in Figure 3b. Besides, the geodesic distances from all vertices to each seed k were calculated and represented by a distance vector ${\text{d}}_{k}$. Given the seed magnitude $a_{k}$ that represents the elevated SUVR value caused by tau pathology, and assuming that the SUVR values exhibit a decay that is proportional to the distance ${\text{d}}_{k}$ from seeds to other vertices, a smooth synthetic SUVR map on the surface can be calculated as

$$\begin{array}{l} \begin{array}{ccc} S\hat{UV}\text{R} & =SUVR_{tem} & \end{array}\\ \;\;\;\;\;\;\;\;\;\;\;\;\;\;\;\;\;\;\;\;\;+\sum_{k=1,2,3}\frac{f({\text{d}}_{k}|0,\sigma_{k})-min(f({\text{d}}_{k}|0,\sigma_{k}))}{max(f({\text{d}}_{k}|0,\sigma_{k}))-min(f({\text{d}}_{k}|0,\sigma_{k}))}\times a_{k}, \end{array}$$ (8)

where f is the probability density function of a Gaussian distribution with zero mean and standard deviation $\sigma_{k}=max({\text{d}}_{k})\text{​}/\text{​}2$ . By sampling the seed magnitudes according to the four different schemes in Figure 3b, four synthetic datasets were generated (Synthetic-1, -2, -3, -4), where each dataset contains 300 SUVR maps for each subtype.

## 3 Experimental Results

### 3.1 Performance evaluation with synthetic data

**Fig. 4.**
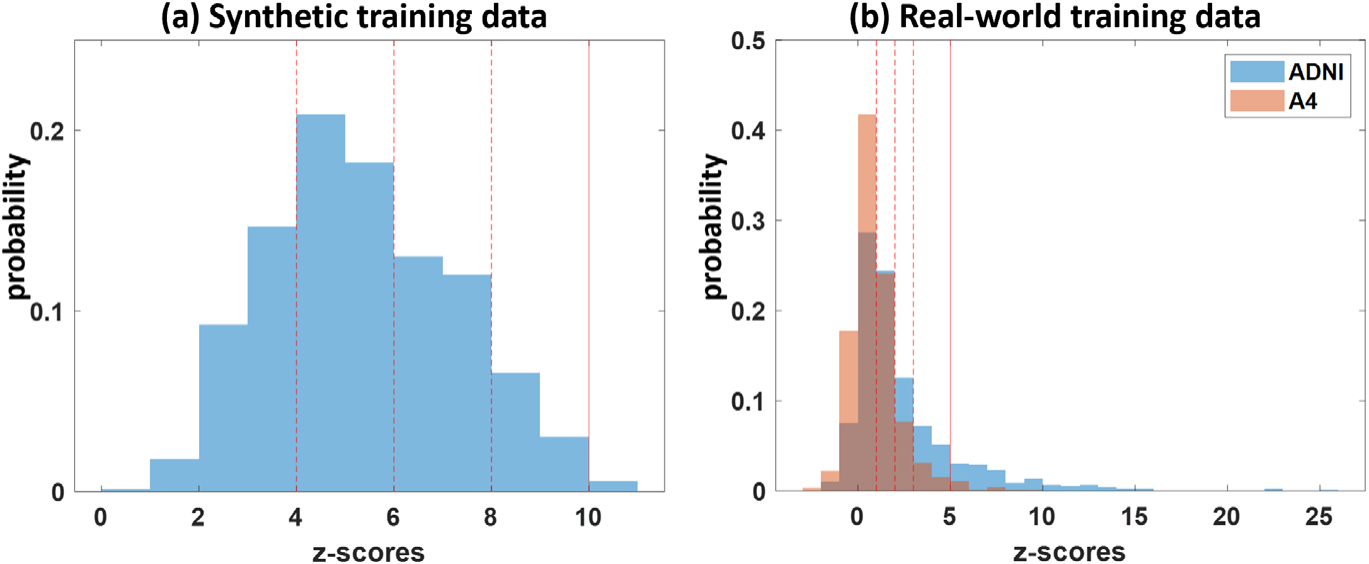
The distribution of the z-scores across all ROIs in the training data. (a) Synthetic-1 training data. (b) Real-world training data. Three z-score values (dashed lines) and a maximum z-score parameter (solid line) are used in even definition for the SuStaIn method.

In the experiments, we trained both our method and SuStaIn on the Synthetic-1 data, and used Synthetic-2, -3, -4 as three different testing datasets. To prepare the input data for the SuStaIn method, we follow the setting in Vogel et al. (2021) by taking the frontal lobe, temporal lobe, medial temporal lobe (MTL), parietal lobe, and occipital lobe as the five ROIs. For each subject, z-scores were calculated at each ROI using the means and standard deviations of the normal controls from the ADNI and A4 cohorts. The z-score distribution of the Synthetic-1 data is shown in Figure 4a. For the SuStaIn method, z-score values of 4, 6, 8 were used for event definition and 10 for the maximum z-score, and the default standard deviation of 1 was adopted to train the SuStaIn model. Subtype assignments for the Synthetic-2, -3, and -4 test datasets were derived from the SuStaIn model likelihoods computed according to the trajectories estimated from the training set. For our method, we directly applied the Reeb graph analysis described in Section 2.3 to all data and constructed the population-level topographical similarity graph using the training Synthetic-1 data by following the procedures outlined in Sections 2.4 and 2.5. The community detection algorithm was implemented with a resolution parameter of γ=0.8 . The subtyping membership of the testing data was subsequently inferred using the KNN-based approach detailed in Section 2.7.

The clustering performance was evaluated using the Adjusted Rand Index (ARI) (Hubert & Arabie, 1985), which quantifies the accuracy of clustering by measuring the similarity between the subtyping results and the ground-truth subtypes. The ARI results of both methods on the four synthetic datasets are given in Table 2. Our method successfully identifies the three subtypes on the training set, and achieves better performance (89.33% ) over the SuStaIn method (82.68% ). Notably, while the ARI of the SuStaIn method decreases on the test sets as the data distribution differences between the training and test sets increase, our method demonstrates stable performance due to its reliance on pattern similarity rather than SUVR values. Furthermore, the performance of our method even slightly increases because of the more apparent patterns caused by the increased seed magnitudes in the testing sets. These results demonstrate the robustness of our method against variations in tau pathology severity between the training and testing sets, and potentially better generalization ability, which will be further demonstrated in experiments on real data from ADNI and A4.

**Table 2.** Subtyping performance on the synthetic data.

ARI (%)	SuStaIn method	Our method
Synthetic-1	82.68	**89.33**
Synthetic-2	77.39	**93.33**
Synthetic-3	75.76	**91.33**
Synthetic-4	75.18	**92.67**

Bold values indicate the best subtyping performance within each row.

### 3.2 Subtyping of ADNI and A4 data

**Fig. 5.**
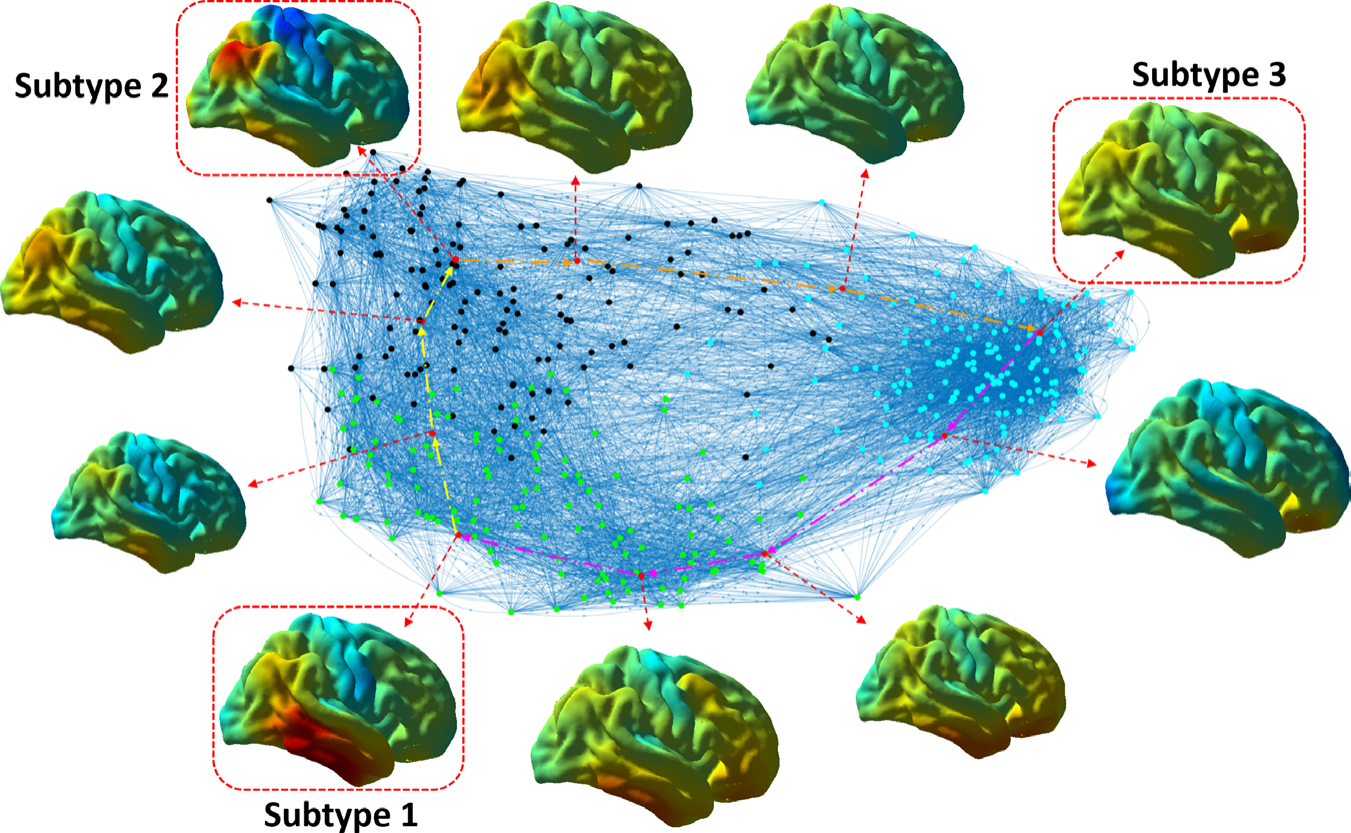
A 2D visualization of the directed population-level graph for the ADNI+A4 data. Green: subtype 1; black: subtype 2; cyan: subtype 3. Tau SUVR patterns of representative subjects for each subtype are visualized.

Using tau PET scans from 427 cross-sectional A+T+ ADNI and A4 subjects as training data in the second experiment, we first construct a directed population-level graph to represent the spatiotemporal tau pathology pattern similarity among these subjects. Based on the observation of the modular distributions in this graphical model (Fig. 5), we take the resolution γ=0.5 for the community detection algorithm to obtain three subtypes, which are consistent with the previous postmortem and neuroimaging findings (Jeon et al., 2019; Lowe et al., 2018; Murray et al., 2011; Whitwell et al., 2012). Subsequently, the disease stages were determined based on the degrees of the nodes in the directed graph (Eq. (7)), and two cutoffs (0 and 10 in this work) were used to define three stages (early/intermediate/late).

The SuStaIn method was applied to the same training data for qualitative comparisons. To prepare the data for the SuStaIn method, we converted the SUVR map of each subject into z-scores of five ROIs as in the synthetic experiments. Based on the z-score distributions as shown in Figure 4b, z-score values of 1, 2, 3 were used for event definition and the maximum z-score was set as 5 for all ROIs in the SuStaIn method. In addition, the σ parameter was set to 0.4 for a robust and separable generation of three subtypes for the SuStaIn method.

A 2D visualization of the population-level graphical representation is shown in Figure 5. The locations of nodes are determined by a force-directed algorithm (Fruchterman & Reingold, 1991) according to the similarity matrix *Si˜m*, which positions more similar nodes closer together while the dissimilar ones further apart. Several representative examples sampled from the graph are shown in Figure 5, where three subjects are highlighted by red boxes with each exhibiting distinct patterns of their corresponding subtypes. The rest intermediate subjects display the pattern progressions across subtypes. Meanwhile, from the population perspective, the densely distributed connections in the graph allow the discovery of subtypes by the community detection algorithm.

**Fig. 6.**
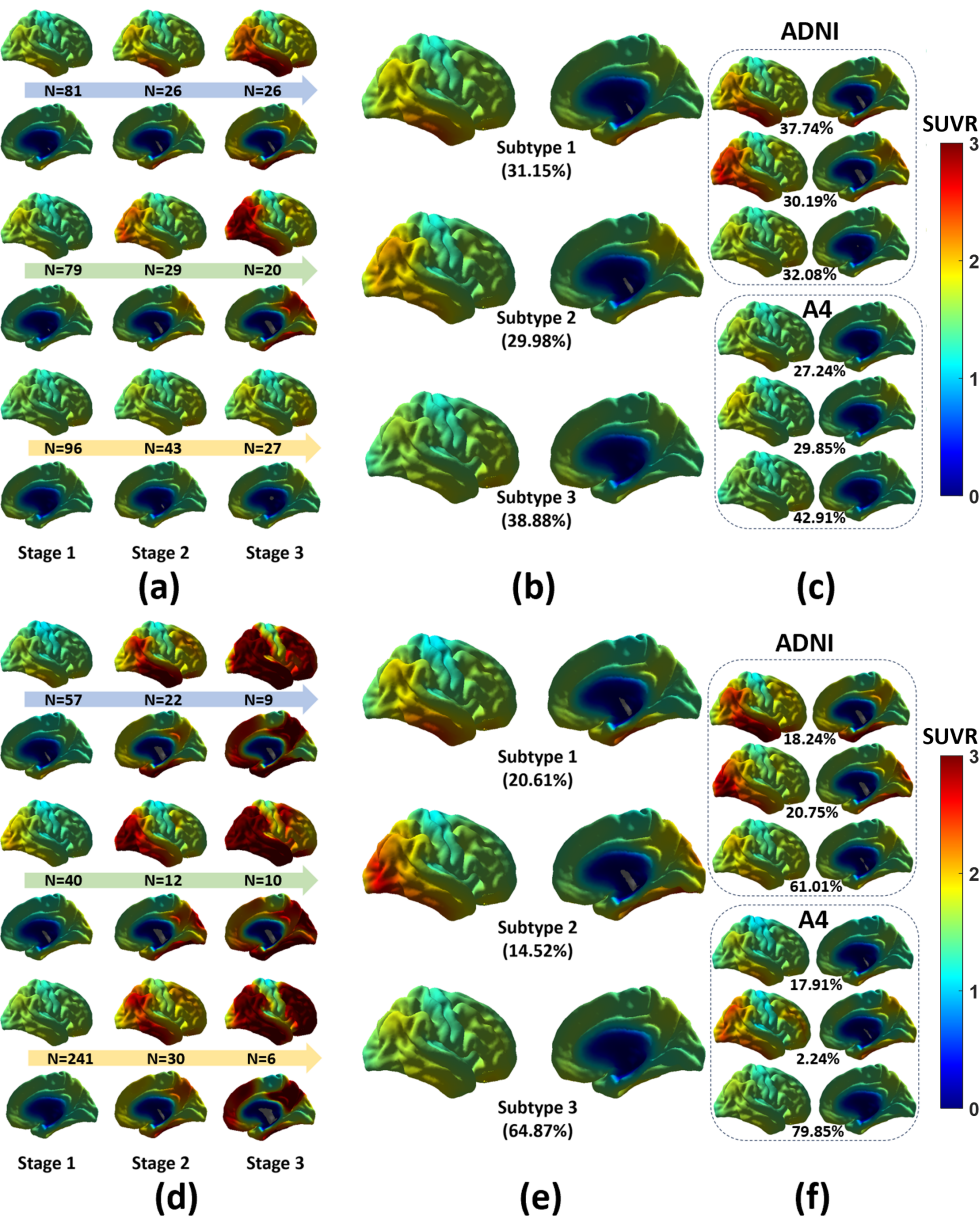
A comparison of subtyping results and progression patterns on the ADNI and A4 data by our method and the SuStaIn method. (a) The average SUVR map of subjects at individual stages of each subtype by our method. Arrangement of the three stages follows the direction of the arrow. N indicates the number of subjects from a particular stage of a particular subtype. (b) The average patterns of subtypes from all training data (ADNI+A4) by our method. (c) The average patterns of subtypes derived separately from the ADNI and A4 cohort by our method. (d) The average SUVR map for every five stages in each subtype by the SuStaIn method. (e) The average patterns of subtypes from all training data (ADNI+A4) by the SuStaIn method. (f) The average patterns of subtypes derived separately from the ADNI and A4 cohort by the SuStaIn method.

The average tau SUVR patterns of each subtype and each stage, along with their respective number of subjects or proportions, were derived from this graphical representation and are shown in Figure 6a and b. In Figure 6a, the progression patterns through three stages for each subtype are plotted in both the medial and lateral view. The tau spatiotemporal patterns show systematic differences across subtypes and consistency along stages within the same subtype. Subtype 1, which contains 31.15% samples, displays a progression pattern consistent with the classic Braak stages (Braak & Braak, 1991), where tau accumulation begins around the entorhinal cortex, subsequently extends throughout the temporal lobe, and finally spreads to the other cortical regions. Subtype 2 contains 29.98% samples, and shows initial tau pathology accumulation in the parietal lobe in stage 1 and subsequently spreads to occipital in stage 2 and other regions in the final stage. Subtype 3, which includes 38.88% samples, is characterized by a moderate uniform tau distribution across the brain regions and a slower SUVR increment along disease stages as compared with the other two subtypes. Within each subtype, the pathology accumulates focally initially and disperses progressively subsequently, maintaining consistent average patterns across stages.

### 3.3 Comparisons of generalization performances

In the third experiment, we evaluated the generalization performances of our proposed method and SuStaIn on subtyping the tau pathology. We first constructed subtyping models using the A+T+ subjects in ADNI (n = 159) and A4 (n = 268) separately for both methods. The model parameters for both methods were the same as in the second experiment on the combined ADNI+A4 cohorts. A qualitative comparison of the three subtypes obtained by the two methods in each cohort is shown in Figure 6c and f. Even with slightly different proportions of each subtype, the average patterns are largely similar between the matching subtypes across the two cohorts for each method. This indicates both methods could replicate the subtyping results in different cohorts.

Furthermore, we assessed the generalization performance of each method quantitatively. More specifically, we applied the model learned from one cohort (training) to a different cohort (testing) and compared the subtyping membership of the testing set with those obtained from the model trained directly on the testing cohort itself. We then used ARI as the metric to quantitatively measure the subtyping similarity between two models within each cohort. The same evaluation process was applied to both our method and the SuStaIn method, and the results are listed for the ADNI and A4 cohort in Table 3. These results show that our method achieves better generalization performances with much higher ARIs when we applied the model learned from one training cohort to an independent testing cohort.

**Table 3.** Generalization performance comparisons.

ARI (%)	SuStaIn method	Our method
A4	71.26	**96.02**
ADNI	70.27	**92.51**
Early-stage subset (SUVR *<* 2)	65.02	**90.58**
Late-stage subset (SUVR *>* 2)	69.08	**93.45**

Bold values indicate the best generalization performance within each row.

To further demonstrate the enhanced generalization performance of our method over SuStaIn is mainly driven by the different ability of each method in handling varying data distributions, we repeated the same generalization experiment between the early- and late-stage A+T+ subjects in the combined ADNI+A4 cohort. For this purpose, we divided the entire set into two subsets according to the peak SUVR values of each subject using a cutoff threshold of 2. Subjects with peak SUVR > 2 were categorized into the late-stage group (n = 270), while the remaining subjects belonged to the early-stage group (n = 157). For both our method and SuStaIn, separate subtyping models were constructed from each group, and the ARI between subtyping memberships obtained from the two models is shown in Table 3 for both the early-stage and late-stage subjects. Using our graphical model, the ARI is 90.58% for early-stage subset and 93.45% for late-stage subset, which indicates the patterns discovered by the topographical modeling are robust to the SUVR magnitude changes. However, similar to results on synthetic data and those between ADNI and A4 cohorts, the SuStaIn method does not have a stable performance (ARI = 65.02% for early-stage subset and ARI = 69.08% for late-stage subset) in the presence of significant magnitude differences between training and testing data.

### 3.4 Validation on ADAD patients

**Fig. 7.**
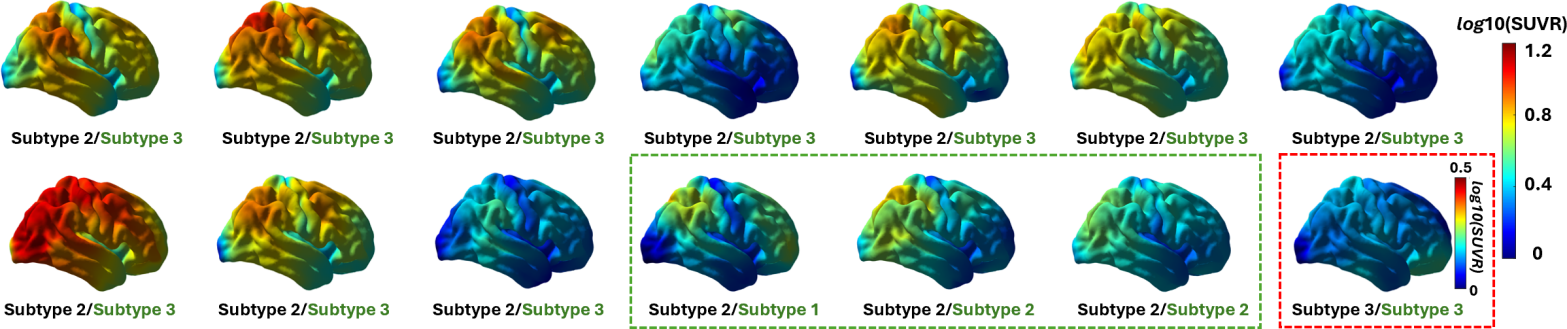
The tau SUVR maps and subtyping results of the 14 ADAD subjects. The logarithm is taken for a better illustration of high SUVR values. The subtyping membership derived from our method (black) and the SuStaIn method (green) is listed under each subject, and the subjects clustered into the minor subtype are highlighted within a red box for our method and green box for the SuStaIn method.

In the fourth experiment, the efficacy of our method was validated with a cohort of 14 T+ ADAD patients, who are carriers of the A431E gene mutation and typically exhibit a consistent and high tau accumulation in the parietal lobe. The SUVR maps of these 14 ADAD patients are shown in Figure 7. The graphical model used for estimating the subtypes was constructed from the combined A+T+ subjects of ADNI and A4 in the second experiment, and the subtyping memberships of ADAD subjects were estimated using the KNN method based on the spatiotemporal similarity between the ADAD patients and all training data in the graphical model. From the experiment, 13 out of 14 ADAD subjects were successfully categorized into Subtype 2 as characterized by the predominant parietal–occipital patterns shown in Figure 6a. While one subject was categorized into Subtype 3, we can see clearly that its tau SUVR map shows a high uptake in the orbitofrontal lobe as compared with other lobes. The SuStaIn method learned from the combined ADNI and A4 data was also applied to the ADAD subjects. As shown in Figure 7, for the SuStaIn method, Subtype 1 and Subtype 2 were minor subtypes with one and two subjects separately, and the majorities were assigned to Subtype 3. The lower stability of the SuStaIn method is primarily due to the extremely high tau SUVR values of ADAD subjects relative to the training data. Overall these results further demonstrate that our method has more robust generalization performance and successfully captures the salient tau pathology patterns of these ADAD subjects determined by their genetic mutations.

### 3.5 Cognitive differences across subtypes in preclinical subjects

In the fifth experiment, we validated our subtyping method by examining the cognitive differences of subjects between subtypes within the A4 cohort, which consists of preclinical subjects with a global cognitive dementia rating (CDR) equal to zero. Three clinical scores were used in our experiments: (1) Mini-Mental State Examination score (MMSCORE) that measures the overall cognitive decline; (2) DIGITTOTAL, the total score of the Cognitive Test—Digit Symbol Substitution Test (COGDIGIT) (Jaeger, 2018), which is used to evaluate associative learning ability; (3) LIMMTOTAL, which is calculated as the immediate recall total score of the Cognitive Test—Logical Memory (Gavett et al., 2016). For all of these measurements, a lower score indicates a more severe impairment in the corresponding cognitive function. For our method and the SuStaIn method, the subtypes of A4 data were obtained in three different subtyping scenarios: (1) the subtypes of A4 data were derived from the model trained with the combination of A+T+ ADNI+A4 data; (2) the subtypes were learned from the model trained with A+T+ A4 data; (3) the subtypes of A4 data were estimated from the model trained with A+T+ ADNI data.

**Fig. 8.**
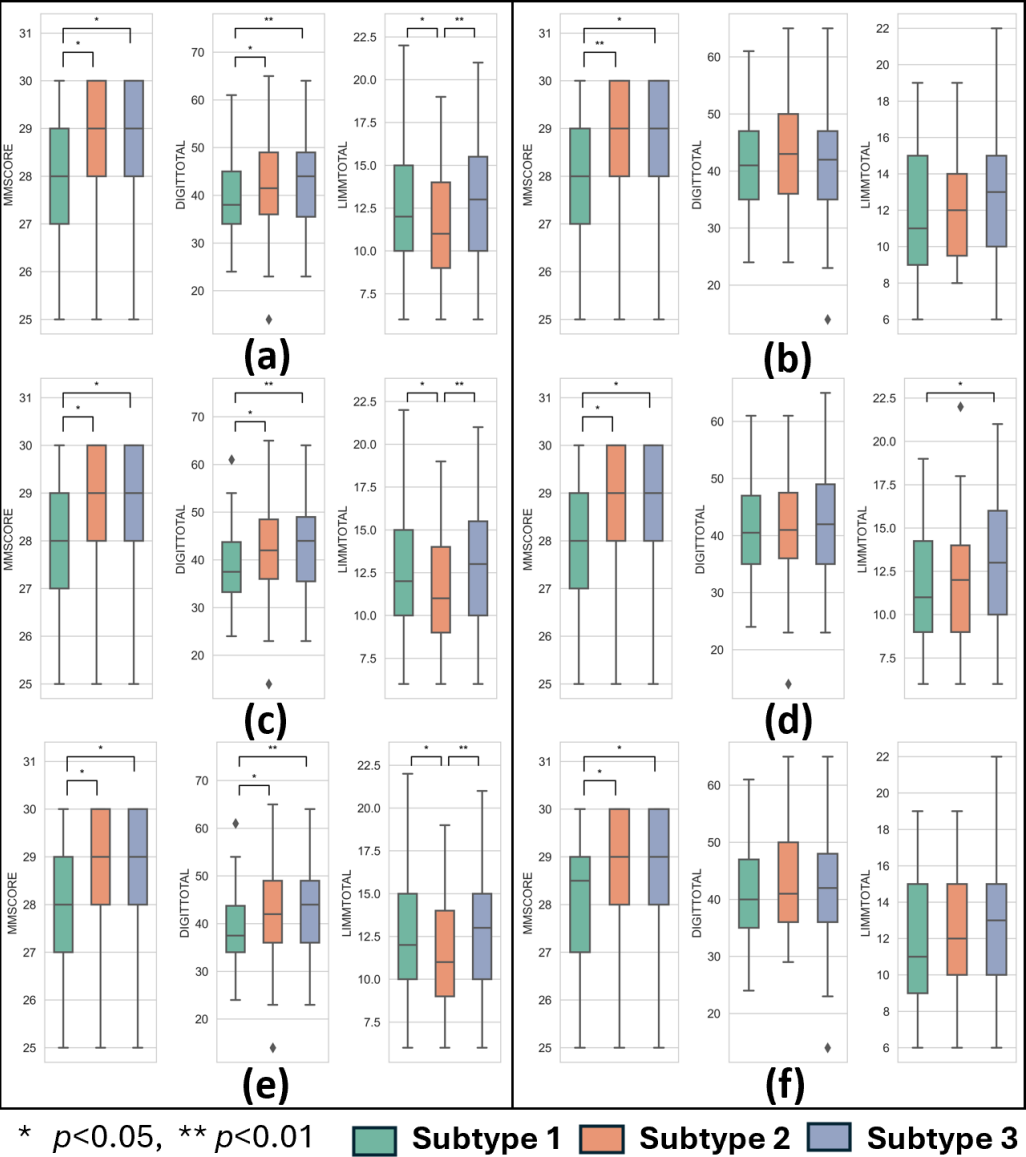
Distribution of three clinical scores (MMSCORE, DIGITTOTAL, LIMMTOTAL) in subtypes of tau pathology in preclinical A4 subjects under three subtyping scenarios. (a) Scenario 1: our method; (b) Scenario 1: SuStaIn; (c) Scenario 2: our method; (d) Scenario 2: SuStaIn; (e) Scenario 3: our method; (f) Scenario 3: SuStaIn.

As shown in Figure 8a, c, and e, there are statistically significant differences in cognitive functions across the subtypes obtained by our method within each subtyping scenario. Subtype 1, which is characterized by a Braak-staging like pattern, has the lowest MMSCORE and DIGITTOTAL scores. This indicates this subtype overall has the worst cognitive decline and impairments in associative learning ability compared with other subtypes. Furthermore, Subtype 2, which exhibits predominant tau pathology patterns in the parietal–occipital lobes, has significantly lower LIMMTOTAL scores and shows the worst logical memory performance as compared with other subtypes. We can also note the distributions of the cognitive functions are almost identical across the corresponding subtypes in all subtyping scenarios using our method. This is due to the stable subtyping results by our method with its robust generalization performances. In contrast, statistically significant differences in cognitive functions are not common across subtypes derived by the SuStaIn method in all scenarios (Fig. 8b, d, f), which indicates this method may not be as sensitive to preclinical data as our method. The weak generalization performance of the SuStaIn method can be further observed from the varying distributions of the DIGITTOTAL and LIMMTOTAL scores in corresponding subtypes obtained under different scenarios. These results indicate that even at the preclinical stage, specific functional changes may already occur among different tau pathology subtypes, whose distinct tau pathology patterns can be successfully detected by our method even at low SUVR values. This suggests the high potential of considering tau pathology phenotypes for the prognosis of AD at the early preclinical stage.

## 4 Discussion

In this paper, we presented a graphical representation for characterizing the cortical topographic patterns of tau pathology, which serves as features for robustly discovering the spatiotemporal heterogeneity within AD. The proposed Reeb graph analysis framework attempts to identify the underlying topographical changes of tau pathology on cortices, which encodes the individual variations and reveals coherent patterns that are shared among subsets within large populations. The spatial and temporal similarity measurements between the graphical representations from different subjects contribute to a directed population-level graph that represents the spatiotemporal distributions in the population, which seamlessly integrates clustering and staging. The subtyping of tau pathology is solved by applying an efficient graph-based community detection algorithm, and the staging of tau pathology is achieved by thresholding the edge degree differences within each subtype.

In our experiments, we demonstrated the ability of the proposed approach to discover subtypes with distinct tau pathology patterns in both synthetic and real-world datasets associated with AD. We used data from ADAD patients with consistent tau pathological patterns to validate the efficacy of the proposed method and its advantage over the state-of-the-art SuStaIn method. Moreover, our method exhibits improved generalization performance because of its focus on topographic patterns of tau SUVRs and the resulting robustness to variations in tau SUVR feature distributions across cohorts. This attribute enables its successful application in the A4 cohort consisting of preclinical subjects without substantial tau pathology, where more significant differences in clinical cognitive measures can be observed between subtypes detected by our proposed method.

### 4.1 Tau pathology heterogeneity in AD

Applying the proposed framework to tau PET imaging data from the ADNI and A4 dataset resulted in the identification of three AD subtypes. The agreement of results in all training datasets in our experiments emphasizes the fact that tau pathological progressions exhibit heterogeneity in AD. The first subtype shows the consistent tau distribution patterns as Braak staging (Braak & Braak, 1991), which is similar to the typical AD group in Murray et al. (2011) and medial–temporal subtype in Jeon et al. (2019). This subtype also exhibits a similar clinical profile of typical AD, and its difference to the other two subtypes can be identified in the preclinical stage (CDR = 0). The second subtype is characterized by the most tau pathology accumulation in the parietal and occipital lobe, which is similar to the parietal-predominant subtype discovered in Jeon et al. (2019). The A431E gene mutation carriers that exhibit a high tau accumulation in the parietal lobe are successfully identified in this subtype. For this subtype, we observe in the A4 cohort that the logical memory scores (LIMMTOTAL) declined more significantly than the other two subtypes at the preclinical stage. The third subtype shows minimal and uniform tau accumulations across the whole brain, and demonstrates the most stable cognitive performance among all subtypes in the A4 cohort.

Our results agree well with previous studies that used tau PET imaging data to analyze AD heterogeneity (Jeon et al., 2019; Lowe et al., 2018) and are similar to the subtypes identified in pathological studies based on the distribution and density of neurofibrillary tangles (Murray et al., 2011). In particular, comparing our subtyping results of the same method with the previous study using the SuStaIn method (Vogel et al., 2021), the general subtype patterns are similar enough to help confirm our results: our first subtype mostly aligns with the limbic-predominant subtype; our second subtype corresponds to the posterior subtype. Although our third subtype differs from the SuStaIn subtypes reported in Vogel et al. (2021), this discrepancy likely arises from the different training dataset used in our work that could include more uniformly and slower progressing subjects. Moreover, despite differences in proportions and SUVR values, our results are consistent with the patterns discovered by the SuStaIn method in our experiments when using identical training data.

### 4.2 Monotonicity assumption for cross-sectional data

While the three subtypes identified in our analysis capture major spatiotemporal patterns of tau pathology, heterogeneity in tau PET extends well beyond topographical spread alone. In practice, regional tau PET signal reflects a combination of disease-driven accumulation and multiple imaging- and biology-related factors that influence the magnitude, dynamic range, and apparent ceiling of SUVR values. These effects can introduce substantial variability even within the same subtype and may partially violate the monotonic accumulation assumption when modeling cross-sectional data.

One substantial source of variability in tau PET measurements arises from differences in the binding properties of tau PET tracers. Tau tracers vary in their affinity for tau aggregates with distinct conformations, maturational states, and isoform compositions, which can produce region-dependent differences in apparent SUVR that are not solely attributable to disease progression (Moscoso et al., 2022). For example, second-generation tau tracers (e.g., PI-2620 and MK-6240) generally exhibit higher specificity and sensitivity to tau pathology than first-generation tracers such as AV-1451, often resulting in increased detectability of tau signal in earlier Braak stages. Consequently, regional SUVR values may differ systematically across tracers even in the absence of true pathological differences. One potential strategy to mitigate these tracer-related effects is the application of harmonization techniques that operate at the voxel or surface-vertex level, which could account for local intensity distortions, thereby providing a more faithful characterization of regional tau PET signal heterogeneity (Yue et al., 2025).

Another imaging factor resulting in regional ceiling variation is off-target binding in regions such as the basal ganglia, choroid plexus, and meninges (Choi et al., 2018; Ikonomovic et al., 2016). Such off-target binding can inflate apparent SUVR values and introduce additional heterogeneity across subjects by creating region-specific differences in attainable tau PET signal beyond topography of tau pathology. To mitigate these artifacts, we have developed a systematic preprocessing pipeline (Yue et al., 2025) for accurately mapping tau-PET signal onto the cortical surface, with the explicit goal of reducing off-target contamination and improving the surface-based SUVR quantification.

Beyond imaging factors, biological heterogeneity plays a critical role in shaping regional tau PET profiles. Regional vulnerability to tau pathology is influenced by cellular composition, molecular properties, and microenvironmental factors, leading to systematic differences in how much tau accumulates locally (Grothe et al., 2018; Leng & Edison, 2021; Torok et al., 2025). Moreover, tau propagation is constrained by large-scale brain connectivity, but regional susceptibility modulates the extent to which propagated pathology accumulates and stabilizes within a given area (Raj et al., 2012; Vogel et al., 2020). As a consequence, tau PET signal may stabilize or vary across regions despite similar inferred disease stages, reflecting regional saturation or partial die-out of tau pathology rather than continued monotonic accumulation. Such effects introduce apparent region-specific SUVR ceilings and represent a potential violation of the monotonicity assumption underlying cross-sectional modeling approaches.

Together, regional differences in attainable SUVR levels arising from imaging and biological heterogeneity may violate the monotonicity assumption underlying the present model, which poses a challenge for cross-sectional inference based on monotonic accumulation. We, therefore, acknowledge this as a potential limitation of the current work. Future extensions of this framework could explicitly address these effects by integrating longitudinal tau PET data, which would enable the estimation of within-region accumulation trajectories. In addition, incorporating region-specific constraints, such as adaptive SUVR ceilings and regional scaling parameters, may further improve the robustness of subtype modeling. These directions offer a path toward more accurate and mechanistic characterization of tau progression in heterogeneous populations.

### 4.3 Parameters selection

A number of parameters were involved in both the model design and the experimental implementation, and their selection can influence the robustness of the graph construction and the accuracy of subtype discovery. The parameters associated with Reeb graph analysis depend primarily on the structure of the cortical surface and the complexity of the SUVR maps. In our experiments, we used a common cortical template, specifically fsaverage_sym , and the SUVR maps were smoothed to achieve a similar complexity level, resulting in approximately 100 retained critical points. Under these conditions, the SUVR map complexity and the cortical surface geometry were well aligned across participants. Therefore, the same parameters could be applied consistently across all experiments and datasets. In the subsequent Reeb graph simplification step, we adopted a persistence threshold δ=300 , which typically yields a pruned Reeb graph with less than 10 edges. This level of complexity provides sufficient resolution to capture meaningful topological changes on the cortical surface while avoiding overly trivial or noisy accumulations of tau pathology. The detailed selection of these parameters is discussed in the corresponding sections of the Methods (Section 2). For future applications of the method to different datasets and cortical surfaces, an appropriate scaling factor should be considered to adjust the parameters accordingly. Because Reeb graph construction and simplification are sequential procedures, the inherent complexity of the initial graph affects the choice of persistence threshold. However, the entire framework is designed to preserve topographical consistency. As a result, the final extracted features should reliably capture the underlying topography of tau pathology on the cortical surface regardless of the specific scale or number of retained biomarkers.

In the similarity measurement, one key parameter is α in Eq. (5), which balances the contributions of the temporal inconsistency penalty $D_{crossing}$ and the spatial incoherency penalty $D_{unpair}$ . The choice of α is determined by the relative magnitudes of these two penalty terms to ensure that neither dominates the similarity score. As a result, according to our observation of the magnitude distributions of two penalty terms, we used α=2 in our experiments. Besides, two additional thresholds, $thd_{\text{pos}}$ and $thd_{\text{diff}}$ , were included to improve the robustness of the severity measurements. In our work, we set $thd_{\text{pos}}=0.5$ and $thd_{\text{diff}}=1.3$ according to previous biological research (Jack Jr et al., 2017; Weigand et al., 2022), as these values provide appropriate cutoff points for distinguishing notable SUVR differences or tau positivity while mitigating the influence of individual variability or measurement noise. For constructing the directionality of the graph, we used Δm=1,000 across all experiments. This setting produces a population-level directed graph predominantly composed of uni-directional edges, which is a key property necessary for the subsequent staging.

To determine the optimal graph pruning parameter K for the KNN method, we conducted a grid search over K ranging from 5 to 40 with a step size of 5. Our strategy was to find the optimal K that would produce a graph sparse enough to ensure all nodes remain connected as a single component while also maximizing the proportion of uni-directional edges. We selected K=15 for constructing a graph that is sparse enough and preserves the most important connections. Finally, while different selections of K could lead to the variations in the graph structure, the clustering results remain generally stable because the sparse graphs consistently retain the most significant connections.

The resolution γ parameter for the community detection algorithm also plays an important role in our subtyping algorithm. In the synthetic data experiment, γ=0.8 was chosen based on the predefined three subtypes. For the experiments with ADNI and A4 data, the selection of γ=0.5 to produce three subtypes was partially informed by our observations of the data distributions in the graph (Fig. 5). Additionally, this resolution leads to results that align with the subtype definitions in previous research (Jeon et al., 2019; Lowe et al., 2018; Murray et al., 2011; Whitwell et al., 2012), ensuring that our findings can be directly compared with existing studies.

### 4.4 Factors contributing to the improved performance

One advantage of our method is the use of Reeb graph analysis to construct a whole-surface representation for modeling the topographic pattern of cortical tau pathology. Unlike ROI-based features used in other methods, which rely on anatomically defined, rigid boundaries and often lack sensitivity to subtle local SUVR changes because of averaging within large cortical regions, our Reeb graph treats the cortical surface as a continuous function domain and adaptively partitions it based on the topology of the SUVR map. This allows the method to capture fine-grained spatial patterns, including cross anatomical ROI propagation, and topological changes that reflect heterogeneous tau accumulation. Moreover, because Reeb graph patches are defined by the geometry of the SUVR function rather than atlas labels, they create a pathology-driven correspondence across subjects, supporting more robust subtype comparison and interpretation. These whole-surface representations thus enable a richer characterization of cortical tau distribution than traditional region-level approaches.

In addition, the similarity measurement for graph construction in our method focuses more on the phenotypic patterns of tau pathology rather than SUVR values alone, which makes it more effective in capturing relationships between subjects within the same subtype but at different disease stages. This flexibility allows our proposed method to be applied to various populations while maintaining robust generalization performances. In contrast, the predefined severity scores used in the SuStaIn method have a more constraining effect on the disease progression trajectories in the discovered subtypes and hence limit the application of a trained model to diverse populations with different severity levels of tau pathology.

Ultimately, this robustness to variations in SUVR distributions enables the proposed graphical representation based on Reeb analysis to achieve greater generalization power when applied to new samples for the inference of their tau pathology subtype.

### 4.5 Lateralization of heterogeneity between hemispheres

In our current framework, we integrated the two hemispheres by calculating the mean SUVR maps on a symmetric cortical surface. This design aligns with the Braak staging framework (Braak & Braak, 1991), which emphasizes the topographical distributions of tau pathology across cortical regions. Integrating data across hemispheres can also substantially reduce model complexity.

To further examine hemispheric asymmetry in tau distributions, we quantified potential SUVR lateralization using both mean SUVR and peak SUVR across hemispheres. Specifically, we assessed (1) differences in mean SUVR between the two hemispheres and (2) differences in the 90th percentile SUVR between the two hemispheres. In both analyses, hemispheric SUVRs were considered symmetric when their relative difference was within 7% (Timmers et al., 2020), which is used to account for minor fluctuations that may arise from imaging noise, inter-subject registration, or physiological variability. The proportions of participants showing left-dominant, right-dominant, or symmetric tau patterns are summarized in Table 4.

**Table 4.** Percentages of subjects showing higher SUVR in the left hemisphere (LH), right hemisphere (RH), or equal SUVR between hemispheres for each subtype and metric.

	Subtype 1			Subtype 2			Subtype 3		
Metric	LH	RH	Equal	LH	RH	Equal	LH	RH	Equal
Mean SUVR	10.53	20.30	69.17	14.06	25.00	60.94	3.01	4.82	92.17
90th percentile SUVR	15.04	19.55	65.41	17.19	24.22	58.59	6.63	4.82	88.55

Across all subtypes, most participants exhibited comparable SUVR values between hemispheres. Among the three subtypes, Subtype 3 displayed the highest degree of hemispheric symmetry, while Subtype 2 retained modest but noticeable asymmetry even after thresholding, suggesting the presence of subtype-specific lateralization tendencies.

For the subtypes or the specific subjects showing hemispheric asymmetry, using our current method as the foundation, finer-grained hemispheric differences, and potential progression mechanisms between hemispheres could be examined within a hierarchical framework. To be more specific, more refined subtypes distinguished by hemispheric dominance or asymmetric propagation trajectories could be identified within each major subtype, offering deeper insight into individual variability and potentially revealing biologically meaningful distinctions that are not apparent in the symmetric representation.

### 4.6 Computational efficiency comparison

We further compared the computational efficiency between the proposed framework and the SuStaIn model. The total runtime of our method was approximately 2 hours. Specifically, the Reeb graph analysis and simplification required about 15 minutes, the pairwise similarity computation across 427 training subjects took around 1.5 hours, and the subsequent directed graph construction and community detection steps required an additional 15 minutes.

In contrast, the SuStaIn method completed within approximately 15 minutes, including biomarker feature preparation (5 biomarkers) and subtyping into 3 subtypes with a total of 15 events along the disease trajectory. All experiments were executed on a Linux CentOS 7 system using a single CPU (Intel(R) Xeon(R) E5-2670 @ 2.60 GHz).

Compared with the SuStaIn model, our method incurs a higher but still feasible computational cost and, in return, provides substantially improved generalization performance as demonstrated in our experiments.

### 4.7 Limitations and future work

There are some limitations to this work. First, the lack of ground truth does not allow us to quantitatively validate the proposed method. However, on the one hand, we used ADAD patients to demonstrate the definition of a subtype, and demonstrated different cognitive performances across subtypes represented by preclinical subjects. On the other hand, our results are consistent with the existing descriptions of pathological neurodegeneration related to AD (Jeon et al., 2019; Lowe et al., 2018; Murray et al., 2011; Whitwell et al., 2012). For future work, we will use more cognitive measurements from clinical datasets and different clinical variants of AD to further validate our method and the findings. Second, the validation of the discovered pseudo-stages and the exploration of temporal changes in pattern and clinical profiles are limited by the cross-sectional data used in this study. For future work, we will extend the current framework to incorporate longitudinal scans, which would allow further improvement of subtyping stability and provide more robust insights into disease progression patterns.

In the current framework, we integrated SUVR maps across hemispheres on a symmetric cortical surface template. Previous studies (Ossenkoppele et al., 2016; Vogel et al., 2021) reported lateralized asymmetry in tau distributions between hemispheres, however, a reversed lateralization pattern was discovered in replication experiments using an additional cohort in Vogel et al. (2021). This suggests that variability in training data could explain these contrasting results. To align with the Braak staging of tau pathology in AD (Braak & Braak, 1991) and the definitions of subtypes derived from neurofibrillary studies (Murray et al., 2011; Whitwell et al., 2012), where data from two hemispheres are averaged for each region, the integration of SUVR maps across two hemispheres could make our results more directly comparable with established frameworks in tau pathology. Exploring the cross-hemisphere progressions in different subtypes and their associated cognitive performance would be a future direction.

Moreover, we would apply our method to other imaging modalities to develop subtyping methods that characterize different aspects of AD pathology. Integrating amyloid PET imaging data and anatomical imaging data into the current tau PET-based framework would contribute to building a comprehensive imaging-level understanding of the heterogeneity in AD (Jack Jr et al., 2016, 2018). This allows us to explore the interactions among different factors, and have an in-depth test of the entire amyloid cascade hypothesis (Hardy, 2009). Furthermore, integrating additional modalities such as blood-based biomarkers and genetic data would enhance our understanding of the full spectrum of AD progression and validate the phenotypes discovered through neuroimaging modalities.

## 5 Conclusion

In this work, we developed a novel graphical representation based on cortical topographic patterns of tau pathology for heterogeneity analysis. The proposed graphical representation encodes the spatiotemporal similarity of tau pathology between subjects and facilitates the robust detection of tau pathology subtypes. With synthetic and large-scale data from the ADNI and A4 study, we showed that our method outperformed the state-of-the-art SuStaIn method in identifying subtypes and reaching better generalization performance. These advantages of our method were further validated in identifying the salient tau topographic patterns of ADAD patients and the detection of significant cognitive differences across subtypes in the preclinical A4 cohort.

## Data Availability

The scripts and code used in this study are available at https://github.com/Jiaxin-Yue/Tau-PET-Graphical-Model. The brain imaging data from the Alzheimer’s Disease Neuroimaging Initiative (ADNI) and the A4 study used in this study are publicly available.
